# Evaluation of the Safety and Immune Efficacy of Recombinant Human Respiratory Syncytial Virus Strain Long Live Attenuated Vaccine Candidates

**DOI:** 10.1007/s12250-021-00345-3

**Published:** 2021-02-09

**Authors:** Li-Nan Wang, Xiang-Lei Peng, Min Xu, Yuan-Bo Zheng, Yue-Ying Jiao, Jie-Mei Yu, Yuan-Hui Fu, Yan-Peng Zheng, Wu-Yang Zhu, Zhong-Jun Dong, Jin-Sheng He

**Affiliations:** 1 College of Life Sciences and Bioengineering, School of Science, Beijing Jiaotong University, Beijing, 100044 China; 2 National Institute for Viral Disease Control and Prevention, Chinese Center for Disease Control and Prevention, Beijing, 102206 China; 3 School of Medicine and Institute for Immunology, Beijing Key Lab for Immunological Research on Chronic Diseases, Tsinghua University, Beijing, 100084 China

**Keywords:** Human respiratory syncytial virus (RSV), RSV long strain, Live attenuated vaccine, Safety, Protective immunity

## Abstract

Human respiratory syncytial virus (RSV) infection is the leading cause of lower respiratory tract illness (LRTI), and no vaccine against LRTI has proven to be safe and effective in infants. Our study assessed attenuated recombinant RSVs as vaccine candidates to prevent RSV infection in mice. The constructed recombinant plasmids harbored (5′ to 3′) a T7 promoter, hammerhead ribozyme, RSV Long strain antigenomic cDNA with cold-passaged (*cp*) mutations or *cp* combined with temperature-sensitive attenuated mutations from the A2 strain (A2*cpts*) or further combined with *SH* gene deletion (A2*cptsΔSH*), HDV ribozyme (δ), and a T7 terminator. These vectors were subsequently co-transfected with four helper plasmids encoding N, P, L, and M2-1 viral proteins into BHK/T7-9 cells, and the recovered viruses were then passaged in Vero cells. The rescued recombinant RSVs (rRSVs) were named rRSV-Long/A2*cp*, rRSV-Long/A2*cpts*, and rRSV-Long/A2*cptsΔSH*, respectively, and stably passaged *in vitro*, without reversion to wild type (*wt*) at sites containing introduced mutations or deletion. Although rRSV-Long/A2*cpts* and rRSV-Long/A2*cptsΔSH* displayed  temperature-sensitive (*ts*) phenotype *in vitro* and *in vivo*, all rRSVs were significantly attenuated *in vivo*. Furthermore, BALB/c mice immunized with rRSVs produced Th1-biased immune response, resisted *wt*RSV infection, and were free from enhanced respiratory disease. We showed that the combination of *ΔSH* with attenuation (*att*) mutations of *cpts* contributed to improving *att* phenotype, efficacy, and gene stability of rRSV. By successfully introducing *att* mutations and *SH* gene deletion into the RSV Long parent and producing three rRSV strains, we have laid an important foundation for the development of RSV live attenuated vaccines.

**Electronic supplementary material:**

The online version of this article (10.1007/s12250-021-00345-3) contains supplementary material, which is available to authorized users.

## Introduction

Human respiratory syncytial virus (RSV) is the most common cause of pneumonia and the key factor in pneumonia-related deaths among children under five years of age. It is also known to cause deaths in the elderly and immunocompromised people (Falsey *et al*. 2005; Jorquera *et al*. 2016; Troeger *et al*. 2018; PERCH 2019). The earlier formaldehyde-inactivated RSV vaccine (FI-RSV) did not prevent RSV infection but resulted in enhanced respiratory disease (ERD) in the vaccinated children (Kapikian *et al*. 1969). Therefore, safety is set as the priority for the development of RSV vaccine for infants, and avoiding ERD as well as stimulating effective immune protection are still the main challenges of RSV vaccine research. One of the promising candidates is the RSV live attenuated vaccine (LAV), which has been successfully obtained by introducing mutations or/and deleting non-essential genes from the RSV genome (Connors *et al*. 1995; McFarland *et al* *.* 2018; McFarland *et al*. 2020a; McFarland *et al*. 2020b). Among the dispensable genomic regions are genes encoding small hydrophobic protein (SH), nonstructural proteins (NS), and M2 ORF protein 2 (M2-2) proteins. The deletions of NS1, NS2, or M2-2 are known to be promising strategies for obtaining intranasal RSV LAV against neonatal RSV infection (Luongo *et al*. 2013; McFarland *et al*. 2020b; Teng *et al*. 2020).

RSV LAV originating from the RSV Long parent strain has not yet been obtained; however, the RSV A2 parent has been widely used to construct RSV LAV candidates (Huang *et al*. 2009; Hu *et al*. 2014; Xu *et al*. 2018; Bouillier *et al*. 2019). RSV Long and A2 strains belong to the same GA1 clade and RSV group A. Moreover, their attachment glycoproteins (G) share 94% sequence identity (Johnson *et al*. 1987; Peret *et al*. 1998). Compared with the G protein, the fusion glycoprotein (F) is even more highly conserved among A and B RSV groups. A few RSV vaccine candidates have been developed by neutralizing antigenic F or G proteins from the RSV Long strain (Singh *et al*. 2007; Huang *et al*. 2009; Magro *et al*. 2012; Blanco *et al*. 2014). RSV LAV candidates from the RSV A2 parent have also been developed; however, different laboratory stocks of RSV parents differed in the level of attenuation (Lawlor *et al*. 2013; McFarland *et al*. 2018). Hence, we aimed to develop RSV LAV candidates from the RSV Long parent using a reverse genetics technology platform and determine whether their efficacy is comparable to that of RSV LAV from the RSV A2 strain.

The known examples of RSV LAV are mainly attenuated by point mutation, gene deletion, or by their combination. The original set of mutations included five cold-passaged (*cp*) mutations, and the temperature-sensitive (*ts*) point mutations identified by reverse genetics method (Crowe *et al*. 1994a, b, 1995). The replication ability of *cpts*-*248/404 in vitro* was weakened at 37 °C and the virus remained attenuated in infants attending the clinical trials. However, viral replication in the nasal cavity caused serious nasal congestion. Another RSV LAV (rA2*cp248/404/1030ΔSH*) was generated by *SH* gene deletion (*ΔSH*) (Bukreyev *et al*. 1997).

Here, we cloned plasmids containing RSV antigenomic cDNA from the wild-type RSV (*wt*RSV) Long strain harboring *cp* mutations, *cpts* mutations, or *cptsΔSH* mutations, and named them pBRB-RSV-rLong/A2*cp*, pBRB-RSV-rLong/A2*cpts*, and pBRB-RSV-rLong/A2*cptsΔSH*, respectively. Then, we co-transfected each of the constructed recombinant plasmids together with four helper plasmids encoding nucleoprotein (N), phosphoprotein (P), large protein (L), and M2-1 into BHK/T7-9 cells expressing T7 RNA polymerase, and the recovered viral particles were blind passaged in Vero cells. The successfully rescued recombinant RSVs (rRSVs), rRSV-Long/A2*cp*, rRSV-Long/A2*cpts*, and rRSV-Long/A2*cptsΔSH*, were analyzed for their genetic stability by sequencing and for their attenuation (*att*) phenotypes by measuring replication activity on the basis of growth curves. The strains were also assessed for temperature sensitivity by measuring viral titers at different temperatures. Finally, BALB/c mice were intranasally inoculated with each rRSV and subsequently challenged with *wt*RSV. The immunogenicity, safety, and efficacy of these rRSVs were evaluated on the basis of serum antibody levels, neutralizing antibody titers, lung pathology, and viral replication activity in the upper and lower respiratory tracts.

## Materials and Methods

### Cells, Viruses, and Plasmids

HEp-2 cells and Vero cells (ATCC, Rockefeller, MD, USA) were grown in Dulbecco's Modified Eagle Medium (DMEM) (Gibco BRL, Gaithersburg, USA) containing 2 mmol/L l-glutamine and 10% fetal bovine serum (FBS, Gibco, Australia). BHK/T7-9 cells were kindly provided by Professor W. Y. Zhu (CDC, Beijing, China) and maintained in DMEM with 10% tryptose phosphate broth (Sigma, Darmstadt, Germany), 5% FBS, 2 mmol/L l-glutamine (Amresco, Solon, USA) and antibiotics (100 IU/mL penicillin, 10 μg/mL streptomycin, 0.25 μg/mL amphotericin B, and 600 ng/mL hygromycin, Sigma). *wt*RSVs of subgroup A strain Long and subgroup B (WV VR1400, ATCC) were propagated using HEp-2 cells cultured in DMEM supplemented with 2% FBS, l-glutamine (2 mmol/L), antibiotics (40 IU/mL penicillin G, 100 μg/mL streptomycin), and 0.2% sodium bicarbonate. The helper plasmids, pCITE-N, pCITE-P, pCITE-L, and pCITE-M2-1, encoding N, P, L, and M2-1 proteins, respectively, were gifts from Dr. Marie-Anne Rameix-Welti (Unité de Virologie et Immunologie Moléculaires, Université Paris-Saclay, Paris, France).

### Rescue and Identification of Recombinant RSVs

**Fig. 1:**
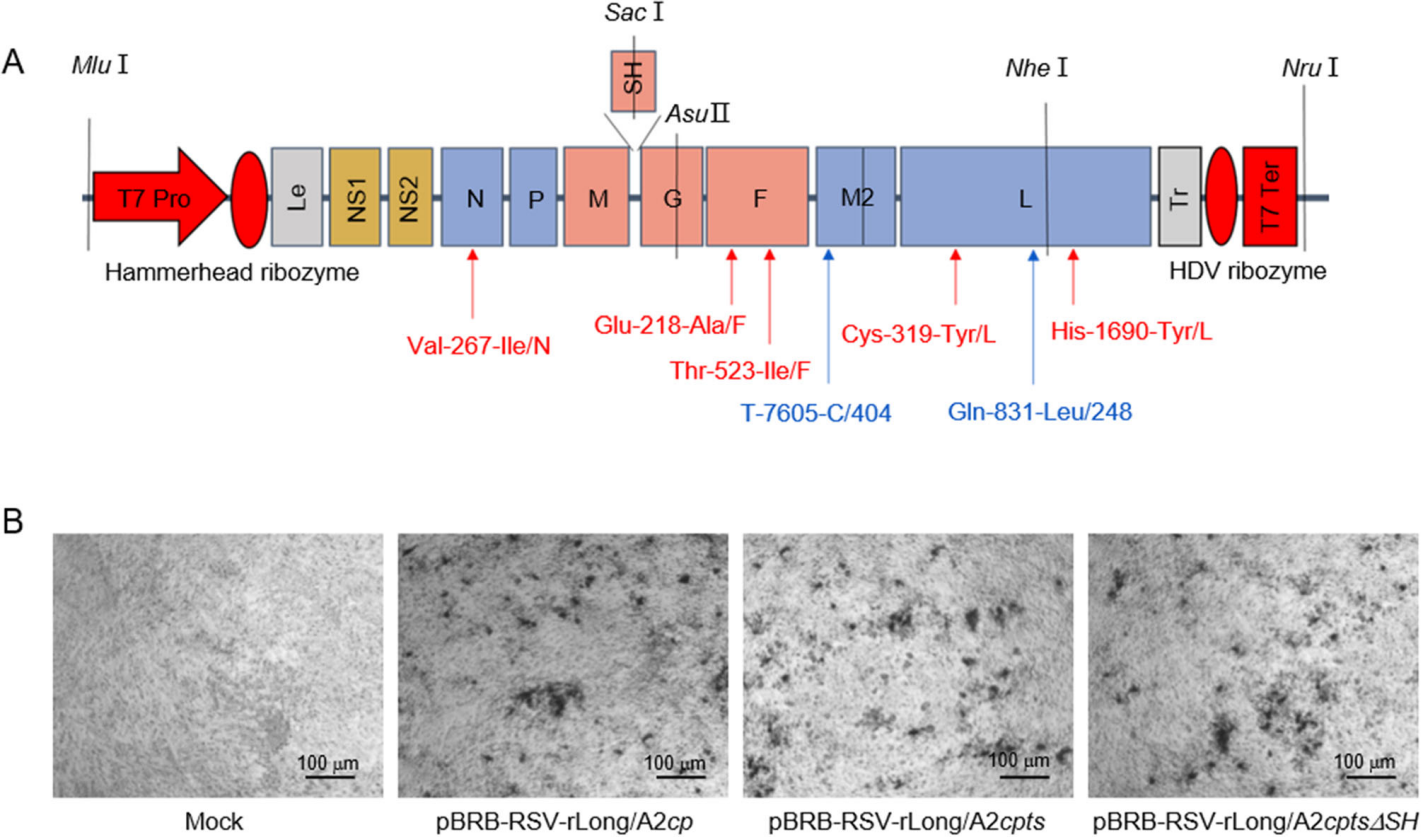
Schematic diagram illustrating our strategy for the identification of recombinant human respiratory syncytial viruses (rRSVs). **A** Schematic diagram of rRSV genomes. The five missense mutations Val-267-Ile/N, Glu-218-Ala/F, Thr-523-Ile/F, Cys-319-Tyr/L, and His-1690-Tyr/L are independent attenuating genetic elements of cold-passaged (*cp*) mutation; Gln-831-Leu/248 and T-7605-C/404 are two independent temperature-sensitive (*ts*) mutations; *ΔSH* is a complete deletion of the *SH* gene. T7 Pro: T7 promoter, T7 Ter: T7 terminator, Le: Lead, Tr: Trail. **B** Identification of replication of the rescued rRSVs by immunoplaque assay. Vero cells were inoculated with suspensions from cells co-transfected with four helper plasmids (Mock), and with pBRB-RSV-rLong/A2*cp*, pBRB-RSV-rLong/A2*cpts*, or pBRB-RSV-rLong/A2*cptsΔSH*, respectively.

The construction and recovery of rRSV-Long/A2*cp*, rRSV-Long/A2*cpts*, and rRSV-Long/A2*cptsΔSH* were performed as reported previously (Xu *et al*. 2018). Briefly, to construct antigenomic cDNA clones, each of the mentioned mutations and deletion was introduced into the full-length *wt*RSV cDNA. Specifically, the deletion included the entire *SH* gene or the majority of the downstream noncoding region of the *SH* gene, as well as silent nucleotide (nt) substitutions in the last three codons and the termination codon of the SH ORF, leaving the gene end signal intact (Bukreyev *et al*. 2001). The resulting plasmids, containing antigenomic cDNAs flanked by T7 promoter-hammerhead ribozyme in the 5′ end and by HDV ribozyme-T7 terminator in the 3′ end (Fig. 1A), were named pBRB-RSV-rLong/A2*cp,* pBRB-RSV-rLong/A2*cpts,* and pBRB-RSV-rLong/A2*cptsΔSH*, respectively. For rescue of rRSVs, BHK/T7-9 cells were co-transfected with a plasmid harboring rRSV antigenomic cDNA and four helper plasmids (pCITE-N, pCITE-P, pCITE-L, and pCITE-M2-1) using Lipofectamine 2000 (Invitrogen, Carlsbad, CA, USA). The transfection mixtures were prepared as follows: rRSV cDNA plasmid 1.25 μg, pCITE-N 1 μg, pCITE-P 1 μg, pCITE-L 0.5 μg, pCITE-M2-1 0.25 μg. The mixtures of DNA-lipo-OptiMEM (Gibco BRL, Gaithersburg, MD, USA) were added into cells after a 20-min incubation. The cells were incubated at 33 °C in a 5% CO_2_ incubator. For the blind passage, Vero cells were incubated with 400 μL of the suspension from the harvested co-transfected cells at 33 °C for 2 h. Then, the infected Vero cells were incubated with DMEM containing 2% FBS at 33 °C and expanded or serially passaged. They were monitored by immunoplaque assay and real-time quantitative PCR (RT-qPCR) as described previously (van Elden *et al*. 2003; Fu *et al*. 2013; Jiao *et al*. 2017). For the immunoplaque assay, serially diluted RSV samples were grown to 80% confluency of HEp-2 cells in a 96-well plate in triplicate for 1 h at 37 °C, and then DMEM containing 0.9% methyl cellulose (Sigma) was added to the wells. Subsequently, the media were discarded and the cells were rinsed with DMEM without FBS. After 3 days of incubation at 37 °C under 5% CO_2_, the cell monolayer was fixed with 95% cold alcohol, and viral replication activity could be assessed using an goat anti-RSV antibody (Millipore, Billerica, MA, USA) incubated with horseradish peroxidase rabbit anti-goat IgG (Santa Cruz Biotechnology, Santa Cruz, CA, USA), and visualized after adding TMB (Promega, Madison, WI, USA). RSV titers were expressed as plaque-forming units per mL (pfu/mL). For RT-qPCR, RNA samples from virus-infected cells were extracted using Trizol reagent (Invitrogen) according to the manufacturer’s instructions, and quantified on the basis of the obtained OD value. The reverse transcription was completed with the GoScript™ reverse transcription system (Promega). Briefly, 1.5 μg RNA samples were incubated with oligo dT for 5 min at 70 °C and then placed for 5 min on ice. Following the addition of 15 μL of RT reaction mix, the samples were placed for 5 min at 25 °C, 1 h at 42 °C, and 15 min at 70 °C, and then stored at 4 °C. Subsequently, qPCR was performed by using a SYBR green probe (Tiangen Biotech, Beijing, China). The primers for the RSV *N* gene were as follows: forward primer, 5′-AGATCAACTTCTGTCATCCAGCAA-3′ and reverse primer, 5′-GCACATCATAATTAGGAGTATCAAT-3′. The thermal cycling conditions included 15 min at 95 °C, followed by 45 cycles of 15 s at 9 °C and 1 min at 60 °C. The specificity of the obtained qPCR products was verified by melting point analysis in the range from 45 to 95 °C.

### Preparation of rRSVs, *wt*RSV, and FI-RSV

The preparation and purification of *wt*RSV and rRSVs were performed in line with a previously described method (Kohlmann *et al*. 2009; Kwanten 2013; Jiao *et al*. 2017). Briefly, the viral samples were inoculated onto HEp-2 cells with 80% confluency and incubated for 3 days at 37 °C under 5% CO_2_. After the formation of syncytia, the cells were scraped off and centrifuged at 3000 rpm for 10 min at 4 °C, and the supernatants were collected. To further release intracellular viral particles, the cell pellet was exposed to three cycles of swift freezing/thawing (the cell pellet was immersed in liquid nitrogen for 10 s and then thawed using running tap water). The frozen/thawed pellet was centrifuged for 10 min at 3000 rpm and the supernatants were collected. The pooled supernatants were run through a 0.45 μm sterile filter (Merck Millipore, Co., Cork, Ireland), and purified by ultracentrifugation on a sucrose cushion gradient (10% sucrose, Sigma) at 17,000 rpm in a P28S rotor (Hitachi, Tokyo, Japan) for 2 h at 4 °C. The pellet was suspended in 300 μL 10% sucrose, divided into aliquots, and stored at  − 80 °C. The infectivity of the RSV was assessed using an immunoplaque assay as described above.

FI-RSV was prepared according to a previously described method (Kim *et al*. 1969). Briefly, RSV-infected cell lysates were clarified by centrifugation for 15 min at 550 ×*g*. Then, the clarified cell lysates were incubated with 37% formalin (Sigma) for 3 days at 36 °C in the proportion of 1:4000. The mixture was then pelleted by ultracentrifugation at 17,000 rpm in a P28S rotor for 1 h at 4 °C. The resulting pellet was resuspended in 1/25 of the original volume in serum-free DMEM and assayed for protein concentration using a BCA protein assay kit (Thermo Fisher Scientific Inc., Waltham, MA, USA).

### *In vitro* Characterization of rRSVs

The above mentioned immunoplaque assay and RT-qPCR were used to evaluate the replication activity and confirm the successful rescue of the rRSVs since passage 1 (P1) in Vero cells. Simultaneously, a GoScript™ reverse transcription (RT)-PCR kit was used to assess the genetic stability of the rescued rRSVs by amplifying fragments of viral DNA harboring the mutated nucleotides contributing to the *att* phenotype. The primers used for RT-PCR are shown in Supplementary Table S1. These characteristic DNA fragments were amplified for each rRSV sample from every odd-numbered passage from P1 to P9 and subsequently sequenced to monitor the possible reversion of individual point mutations.

The rRSV growth kinetics was also assayed using a previously described method (Collins and Bermingham 1999; Schickli *et al*. 2012). Briefly, HEp-2 cells were infected with *wt*RSV or rRSVs at a multiplicity of infection (MOI) of 0.1 in triplicate and incubated at 32 °C. Cells and supernatants were harvested at 24-h intervals post-infection, and viral titers were determined by immunoplaque assay as mentioned above.

The temperature-sensitive phenotype of rRSVs *in vitro* was evaluated by determining the efficiency of plaque formation at various temperatures, through a modified method from a previous report (Crowe *et al*. 1993). Briefly, a HEp-2 cell monolayer, grown in 24-well plates, was inoculated with tenfold serial dilutions of *wt*RSV and rRSV cultures, incubated for 5 days at 32 °C, 35 °C, 36 °C, 37 °C, 38 °C, 39 °C, and 40 °C, and then assayed for infectivity at each corresponding temperature using the immunoplaque assay.

The temperature stress test was performed as reported previously (Collins and Bermingham 1999; Schickli *et al*. 2012). Briefly, rRSVs were passaged at elevated non-permissive temperatures, twice at 37 °C, twice at 39 °C, and once at 40 °C. Next, HEp-2 cells were inoculated with rRSVs at 0.1 MOI in a 96-well plate. Following a 1-h incubation at 33 °C, the inoculum was collected by pipetting, and cells were supplied with 200 μL/well of DMEM with 2% FBS and incubated at 37 °C. Following a 5-day incubation at 37 °C, 100 μL of the media from each of the infected wells were transferred to an uninfected 96-well plate containing HEp-2 cells, in duplicate. After a 1-h incubation, the media were removed by pipetting and the cells were supplied with 200 μL/well of fresh medium. The plates were then incubated for 5 days at 37 °C for the second passage. The virus was similarly transferred and incubated at 39 °C to generate the third and fourth passages. The last passage was performed at 40 °C. For each passage, one of the duplicate HEp-2 plates was immunostained to assess RSV infectivity.

### Replication of rRSVs in the Upper and Lower Respiratory Tracts of Mice

Six-week-old female BALB/c mice (Charles River Laboratories, Beijing, China) were housed in five groups under pathogen-free conditions in microisolator cages at the animal quarters of Tsinghua University Medical Center (Beijing, China). The replication of rRSVs was evaluated in the upper and lower respiratory tracts of mice as previously described (Whitehead *et al*. 1998a). Briefly, mice were infected via intranasal (i.n.) route by rRSVs or *wt*RSV (at 1 × 10^6^ pfu/mouse) and sacrificed by CO_2_ inhalation for washes of nasal and lung tissues on days 2, 4, 6, or 8 post-infection. Viral titers in the harvested washes of nasal and lung tissues were determined using the immunoplaque assay and RT-qPCR as mentioned above. For RT-qPCR, viral RNA extracts from nasal and lung washes were obtained using Trizol reagent according to the manufacturer's instructions, and all the remaining RT-qPCR procedures were repeated as described previously.

Nasal washes were collected as previously reported (Rostad *et al*. 2016). Briefly, the jaws and the head of each sacrificed mouse were disarticulated and sequentially removed. Next, 1 mL of Iscove's Modified Dulbecco's Medium (IMDM) (Gibco, Paisley, Scotland, UK) containing 15% glycerin mixed with 2% FBS-MEM (1:1, vol/vol) was pushed through each naris (total of 2 mL). All nasal washes were collected and stored at  − 80 °C.

The lungs were harvested from mice in each group as previously reported (Fu *et al*. 2014), weighed, placed in phosphate-buffered saline (PBS) containing 1% bovine serum albumin (100 μL/0.1 g lung), and homogenized with a glass tissue grinder. The lung homogenates were centrifuged at 12,000 ×*g* for 10 min and stored at  − 80 °C.

### Animal Vaccination and Challenge

BALB/c mice were randomly grouped (five mice/group), lightly anesthetized with avertin, and then subjected to either i.n. immunization with rRSVs (1 × 10^6^ pfu/mouse) or *wt*RSV (1 × 10^6^ pfu/mouse) or intramuscular (i.m.) immunization with FI-RSV (1.875 μg/mouse) in 50 μL of PBS with 10% sucrose. Alternatively, the mice were subjected to i.m. injection containing only 50 μL PBS with 10% sucrose and were labeled as the negative control group. For the challenge experiments, the immunized or negative control BALB/c mice were further lightly anesthetized and infected with 1 × 10^6^ pfu of *wt*RSV at day 28 after vaccination.

### Evaluation of RSV-Specific Serum IgG Responses by ELISA

Blood samples were collected as previously reported (Fu *et al*. 2014). Briefly, the samples were collected from the retro-orbital plexus of mice with a capillary tube. After centrifugation at 6000 ×*g* for 15 min, the obtained sera were stored at 4 °C. RSV-specific serum antibody responses (Jiao *et al*. 2017; Ma *et al*. 2018) were analyzed by adding tenfold dilutions of serum samples to the RSV-coated plates, which were then incubated for 1 h at 37 °C. Following three washes with PBS, the plates were incubated with HRP-conjugated anti-mouse IgG, IgG1, or IgG2 antibodies (1:5000 dilution, Santa Cruz Biotechnology) for 1 h, developed with 100 μL of TMB substrate solution (Sigma), stopped with 50 μL of 2 mol/L H_2_SO_4_, and analyzed at 450 nm using an ELISA plate reader (Tecan, Grodig, Austria). The titers of anti-RSV antibodies were expressed as the reciprocal of the maximal dilution of serum giving an absorbance reading greater than 0.2 absorbance units and being two-fold above the absorbance obtained for the negative control group.

### RSV-Specific Neutralizing Antibody Assay

To analyze RSV-specific neutralizing antibody titer (Jiao *et al*. 2017), the samples of sera were heat-inactivated at 56 °C for 30 min and prepared by two-fold serial dilution in separate virus diluents. Fifty pfu of RSV virus suspensions (subgroup A *wt*RSV Long or subgroup B (WV VR1400)) were incubated with serially diluted samples for 1 h at 37 °C. Then, the neutralizing antibody titers were determined by immunoplaque assay as described above. Neutralization titers were expressed as the reciprocal of the dilution of serum giving a 50% reduction in the number of pfu in control wells.

### Pulmonary Histology of RSV-Infected Mice

Histological analyses of lung tissues following RSV challenge were performed as described previously (Jiao *et al*. 2017). After mice were sacrificed by CO_2_ inhalation, the lungs were fixed via 4% formalin infusion through the trachea. The solution was removed and the lungs were subsequently immersed in 4% formalin for 24 h, embedded in paraffin, sectioned, and stained with hematoxylin and eosin (H&E). Ten sections of 100 fields per mouse were examined. Sections from each mouse were blindly scored for the degree of pulmonary inflammatory changes including peribronchiolitis, perivasculitis, alveolitis, and interstitial pneumonitis. They were graded as follows: 0, clear; 1, slight; 2, moderate; 3, abundant; and 4, severe.

The eosinophilia in lung tissues of RSV-challenged mice was determined as previously described (Jiao *et al*. 2017). Briefly, the formalin-fixed, paraffin-embedded lung tissue sections were stained with specific stain kits (ZSGB-BIO, Beijing, China), and interpreted by assessment of both staining distribution and intensity with Image-Pro Plus software (Media Cybernetics, Washington, D.C., USA).

### Statistical Analyses

Statistical analyses of data were performed using GraphPad Prism 5 software (GraphPad Software, La Jolla, CA, USA). Differences were evaluated using independent, two-sided Student’s *t* test. Results characterized by *P* < 0.05 were considered statistically significant.

## Results

### Construction and Identification of rRSVs

The plasmids encoding rRSV-Long/A2*cp*, rRSV-Long/A2*cpts*, and rRSV-Long/A2*cptsΔSH* were obtained by a stepwise assembly of the synthesized cDNA segments. The locations of *cp* and *ts* mutations are shown in Fig. 1A. The full-length antigenomic cDNAs of pBRB-RSV-rLong/A2*cp* and pBRB-RSV-rLong/A2*cpts* were both expected to be 18,815 bp in size, while that of pBRB-RSV-rLong/A2*cptsΔSH* was expected to be 18,485 bp in size. The corresponding lengths were confirmed by DNA sequencing (data not shown).

For the recovery of rRSVs, the plasmid containing RSV antigenomic cDNA was co-transfected together with four plasmids encoding helper proteins to BHK/T7-9 cells, and the recovered rRSVs were subsequently blindly passaged in Vero cells. The rescued rRSVs were identified by immunoplaque assay as shown in Fig. 1B and by RT-PCR (data not shown). These results demonstrated that we successfully rescued the rRSVs bearing the anticipated *cp*, *cpts*, and *cptsΔSH* mutations.

### *In vitro Characterization of rRSVs*

**Fig. 2:**
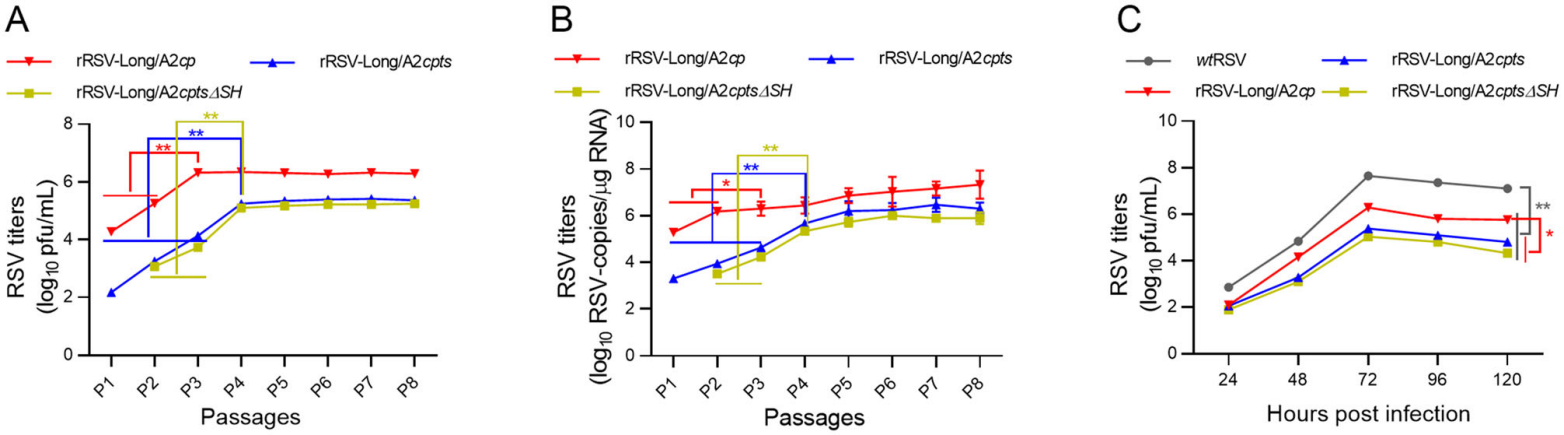
*In vitro* characterization of recombinant human respiratory syncytial viruses (rRSVs). The replication titers during serial passages of rRSVs were monitored by immunoplaque assay (**A**) and by RT-qPCR (**B**) since passage 1 (P1). The growth curves for rRSVs and wild-type RSV (*wt*RSV) were established and compared (**C**). Each virus was harvested every other 24 h post-infection and titers were assayed by immunoplaque assay. All results are representative of three independent experiments. Data are shown as mean ± SD. *P < 0.05, **P < 0.01.

The titers of rRSV-Long/A2*cp* increased rapidly during the initial passages from passage 1 (P1) to P3 (*P* < 0.01) and remained constant after P3 as shown by immunoplaque assay and RT-qPCR. In contrast, the titers of rRSV-Long/A2*cpts* and rRSV-Long/A2*cptsΔSH* increased steadily until P4 (*P* < 0.01), and fluctuated marginally after P4 (Fig. 2A, 2B). The sequencing of the DNA fragments enclosing the individual rRSV point mutations determined the genetic stability of the rescued viruses. All the introduced *att* mutations were stable, and no reversion was detected. The sequencing results for passages from P1 to P9 are shown in Supplementary Table S2. To further characterize rRSVs, the growth kinetics of rRSVs and *wt*RSV in HEp-2 cells at 33 °C were assessed and compared between the two groups. The viral titers began to increase from 24 h post-inoculation and ultimately plateaued at 72 h post-inoculation for all the viruses. *wt*RSV achieved the titer of 4.5 × 10^7^ pfu/mL, rRSV-Long/A2*cp* 2.8 × 10^6^ pfu/mL, rRSV-Long/A2*cpts* 2.2 × 10^5^ pfu/mL, and rRSV-Long/A2*cptsΔSH* 1.1 × 10^5^ pfu/mL, as shown in Fig. 2C. All the three rRSVs exhibited approximately 10- or 100-fold reduced growth kinetics compared with that of the parent strain. Altogether, all rRSVs bearing the *att* mutations were constructed successfully, showed a markedly attenuated phenotype *in vitro*, and their proliferation rates were significantly lower when compared with that of the *wt*RSV parent.

### Analysis of *ts* Mutation Phenotype of rRSVs* In Vivo* and* In Vitro*

The *ts* phenotype of the rRSVs was examined by determining the efficiency of plaque formation by inoculation of tenfold viral dilutions at various temperatures in HEp-2 cells placed in TC24-well plates and comparing the results for *wt*RSV and rRSVs. The plates were incubated for 5 days at the specified temperatures in CO_2_ incubators calibrated to ± 1 °C and the average viral titers were measured by immunoplaque assay in HEp-2 cells at the corresponding temperatures (Table 1). Both rRSV-Long/A2*cpts* and rRSV-Long/A2*cptsΔSH* exhibited reduced viral titers by more than 2log_10_ at 37 °C compared to the permissive temperature of 32 °C and were therefore considered *ts* at ≥ 37 °C. As expected, the *wt*RSV as well as the precursor virus rRSV-Long/A2*cp* were not *ts* and showed no statistical titer reduction from 32 to 40 °C.

**Table 1:** Characterization of the temperature sensitivity and attenuation (*att*) phenotypes of recombinant RSVs (rRSVs) *in vitro* and *in vivo*.

Virus	Mean virus titer^a^ (log_10_ pfu/ml ± SD) at the indicated temperature (°C)							Mean titer in mice^c^	
	32	35	36	37	38	39	40	Nasal wash (log_10_ total pfu ± SD)	Lung (log_10_ pfu/g tissue ± SD)
*wt*RSV	6.9 ± 0.2	6.9 ± 0.1	6.8 ± 0.1	7.1 ± 0.1	7.1 ± 0.1	6.8 ± 0.1	6.8 ± 0.1	3.1 ± 0.1	4.4 ± 0.2
rRSV-Long/A2*cp*	6.1 ± 0.1	6.1 ± 0.1	6.0 ± 0.1	6.0 ± 0.1	5.9 ± 0.1	5.8 ± 0.1	5.7 ± 0.1	2.6 ± 0.1	3.1 ± 0.1
rRSV-Long/A2*cpts*	6.0 ± 0.1	5.2 ± 0.1	4.7 ± 0.1	< *1.0* ^*b*^	< 1.0	< 1.0	< 1.0	2.3 ± 0.1	2.0 ± 0.1**
rRSV-Long/A2*cptsΔSH*	5.4 ± 0.1	4.3 ± 0.2	4.2 ± 0.1	< *1.0* ^*b*^	< 1.0	< 1.0	< 1.0	2.1 ± 0.1	1.9 ± 0.1*

^a^n = 3 replicates (at each temperature).

^b^Shut-off temperature is defined as the restrictive temperature at which a 100-fold or greater reduction compared to the titer observed at the permissive temperature of 32 °C and the lowest shut-off temperatures for each virus are italic.

^c^Groups of five mice were administered 1 × 10^6^ pfu of the indicated virus intranasally under light anesthesia on day 0 and sacrificed on day 4. Virus titer was determined in the nasal washes (log_10_ total pfu) and lung tissues (log_10_ pfu/g tissue).

*The significant difference of RSV replication titers between nasal wash and lung tissue. All the results were shown as the representive of three independent experiments. Data were shown as mean ± SD. *P < 0.05, **P < 0.01.

**Fig. 3:**
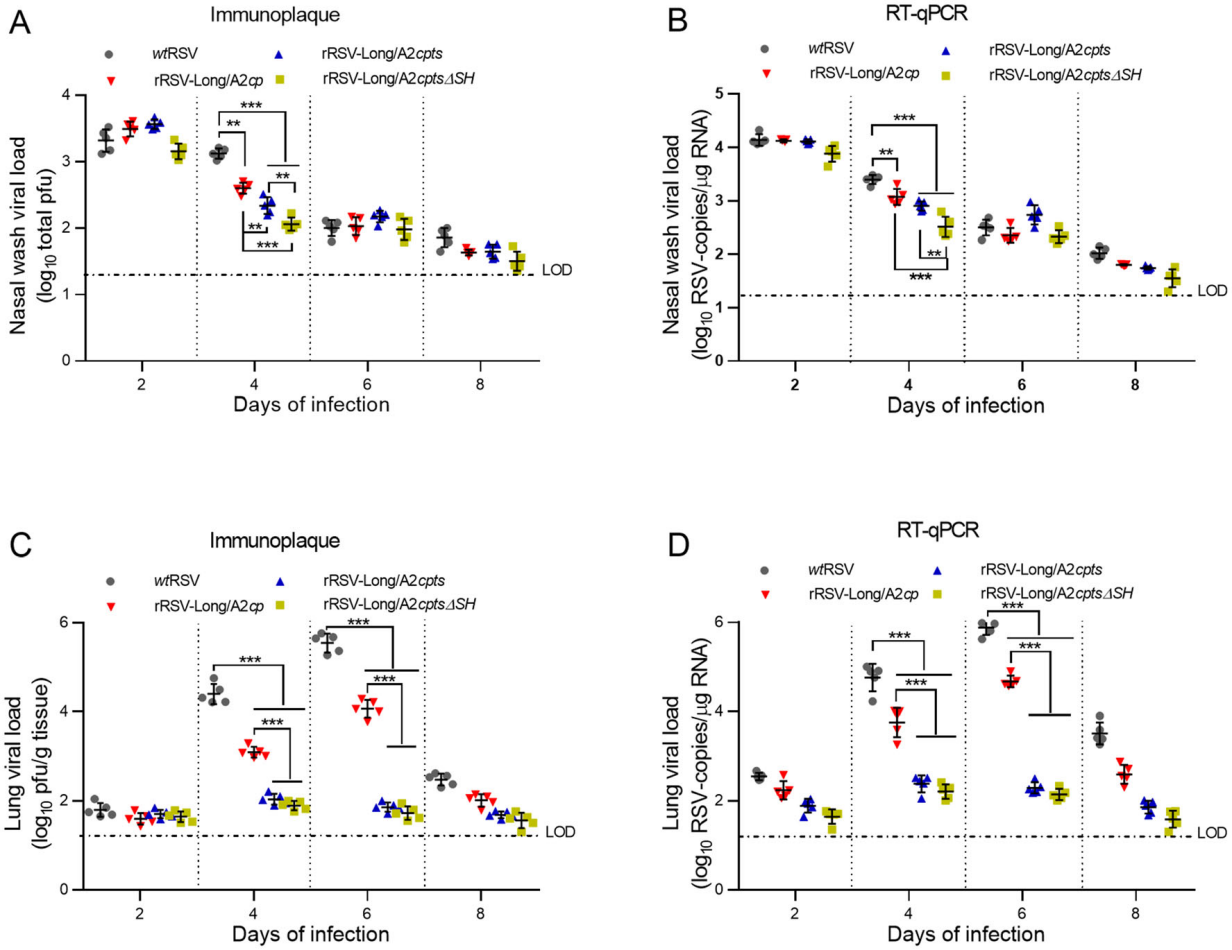
The attenuation (*att*) phenotype of recombinant human respiratory syncytial viruses (rRSVs) assayed in intranasally infected BALB/c mice. Viral titers in samples obtained from nasal washes were analyzed by immunoplaque test (**A**) and RT-qPCR (**B**); viral titers in lung tissues were analyzed by immunoplaque test (**C**) and RT-qPCR (**D**). Data are shown as mean ± SD. **P < 0.01, ***P < 0.001. LOD: limit of detection.

We also intranasally infected mice with 1 × 10^6^ pfu of rRSVs and measured viral titers in samples obtained from nasal washes and lung tissue homogenates on days 2, 4, 6, and 8 post-infection by using immunoplaque assay and RT-qPCR (Fig. 3A–3D). The results showed that the titers of all three rRSVs in the nasal wash specimens at day 4 were 0.52–1.06 log_10_ total pfu, lower than that of *wt*RSV (*P* < 0.01–*P* < 0.001), and the titers in the lung tissues at day 4 and day 6 were 0.51–2.51 and 1.55–3.99 log_10_ pfu/g tissue, lower than the respective ones of *wt*RSV (*P* < 0.001). The number of RNA copies of the three rRSVs in the nasal wash specimens at day 4 was 0.32–0.88 log_10_ RSV copies/μg RNA, lower than that of *wt*RSV (*P* < 0.01–*P* < 0.001); in addition, in the lung tissues at days 4 and 6 the three rRSVs exhibited 1.00–2.55 and 1.20–3.74 log_10_ RSV copies/μg RNA, respectively, lower than the corresponding number of RNA copies of *wt*RSV (*P* < 0.001). These results are consistent with the corresponding immunoplaque test results. Among the three rRSVs, the two rRSVs possessing *ts* mutations displayed a higher level of attenuation than rRSV-Long/A2*cp* in the lung tissues (*P* < 0.001).

We also found that the replication titers of each rRSV bearing *ts* mutations were significantly higher in the samples obtained from nasal washes than in those obtained from lung tissue homogenates (*P* < 0.05 or *P* < 0.01), as shown in Table 1. Consistent with their *in vitro ts* phenotypes, the two rRSVs harboring *ts* mutations displayed shut-off temperatures equal to 37 °C and are therefore more competent to multiply in the nasal cavity environment, characterized by lower temperatures (Table 1).

**Fig. 4:**
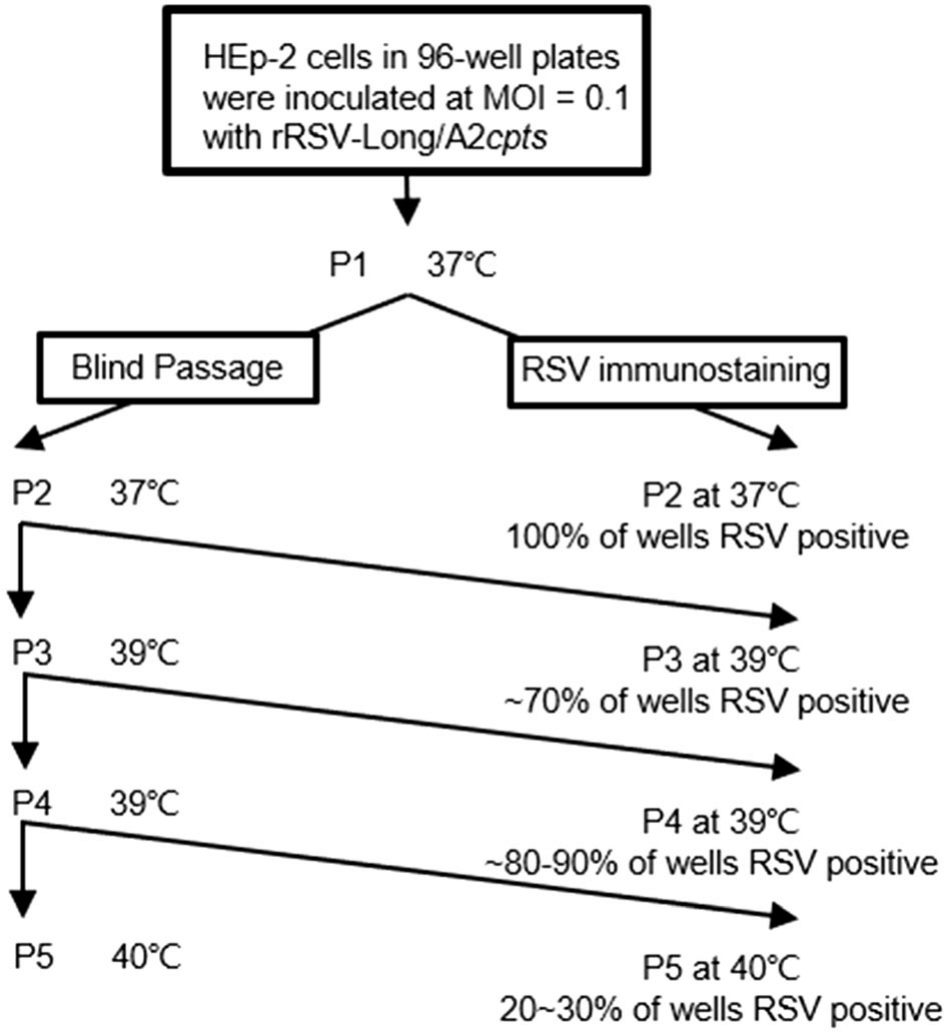
Passaging of rRSV-Long/A2*cpts* at 37 °C and 39 °C. To determine the frequency of intermediate temperature sensitive (*tsi*) viruses, two plates of HEp-2 cells were inoculated at multiplicity of infection (MOI) = 0.1 and supplied with 200 μL of medium. At 5 days post-infection new uninfected HEp-2 cells in 96-well plates were inoculated with 100 μL/well of supernatant from the previous virus passage. Following a 1-h incubation, the inoculum was removed and the cells were fed with 200 μL of medium. Blind passaging was performed in duplicate and at progressively higher temperatures. For each passage, one plate was immunostained and a duplicate plate was used to seed the next passage. Six wells of HEp-2 cells infected with wild-type RSV (*wt*RSV) were used as a positive control.

To determine both the genetic and phenotypic stability of rRSVs, rRSV-Long/A2*cpts* was passaged *in vitro* at non-permissive temperatures to induce mutation, in line with the classical theory of the survival of the fittest. Briefly, rRSV-Long/A2*cpts* was serially passaged twice at 37 °C, and then subjected to two further passages at 39 °C and one passage at 40 °C (Fig. 4). After each passage, one of the duplicate plates was immunostained with anti-RSV antibodies. Initially, after the expansion at 37 °C, all the wells exhibited positive RSV immunostaining. After P4 at 39 °C, more than 80% of the wells had positive RSV immunostaining, suggesting the presence of the temperature-sensitive intermediate (*tsi*) viruses at this temperature. At 40 °C, the control wells containing *wt*RSV all had positive immunostaining. In sharp contrast, only 20%–30% of the wells containing rRSV-Long/A2*cpts* had positive RSV immunostaining. To investigate the nt changes at the *ts* markers, we analyzed the sequence of 1–2 kb cDNA fragments spanning from the start of the *M2* gene through the *L* gene. RT-PCR was performed on five randomly chosen potential *tsi* revertants from RSV-positive wells to detect nt changes at the 248 and 404 *ts* sites of the *L* and *M2* genes by sequence analysis. The characteristics of the biologically derived *tsi* viruses from the passaging of rRSV-Long/A2*cpts* at 39 °C are listed in Supplementary Table S3. The *tsi* strain were characterized by nt changes causing the reversion to the *wt* nt or amino acid at the 248 or the 404 *ts* markers and the partial loss of temperature sensitivity.

### Characteristics of RSV-Specific Immune Responses and Neutralizing Antibody Responses

**Fig. 5:**
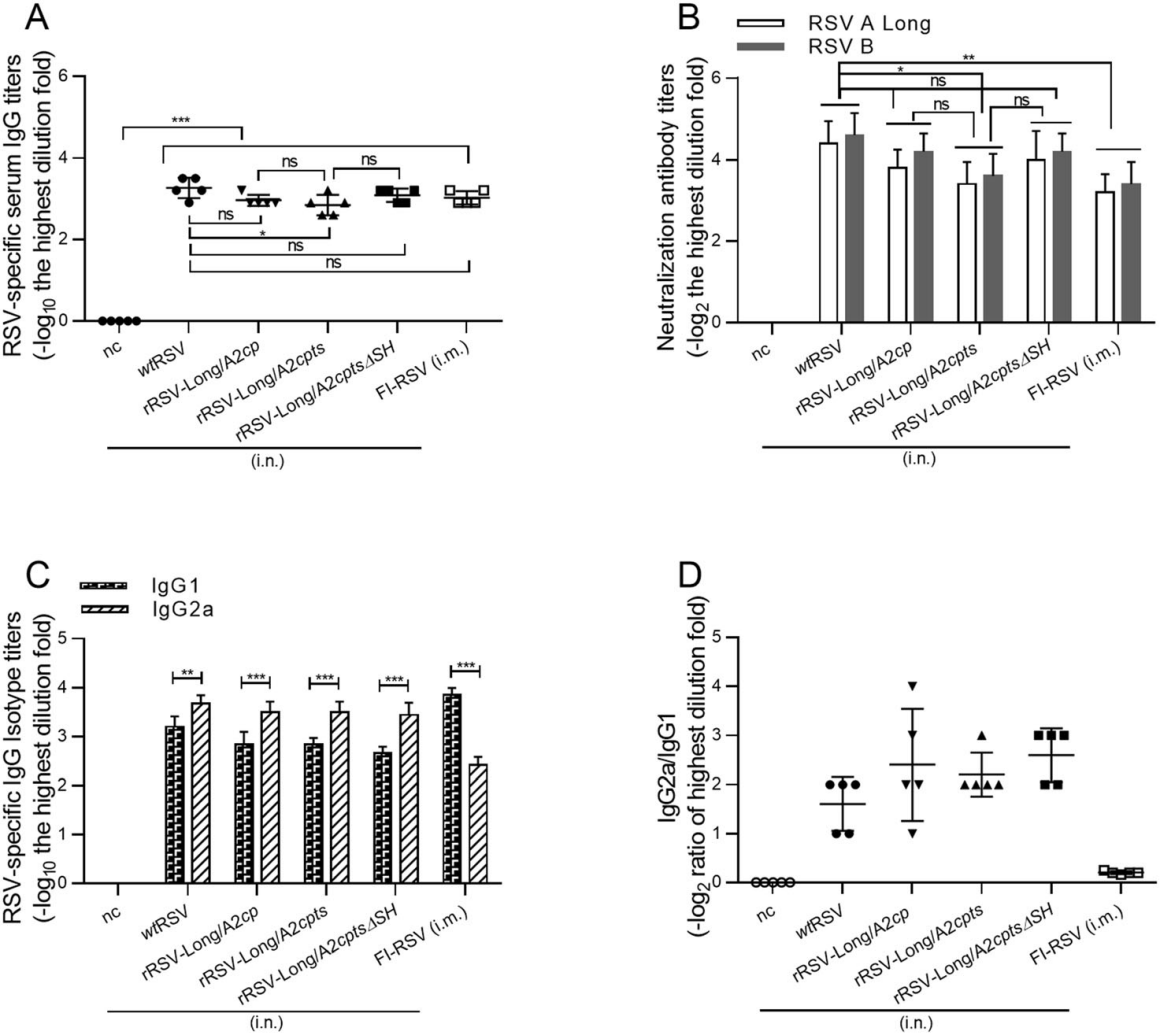
RSV-specific immune responses and neutralizing antibody responses following infection with recombinant human respiratory syncytial viruses (rRSVs) and wild-type RSV (*wt*RSV). BALB/c mice were immunized with either rRSV-Long/A2*cp*, rRSV-Long/A2*cpts*, rRSV-Long/A2*cptsΔSH*, or *wt*RSV at 1 × 10^6^ pfu/mouse or PBS (50 μL/mouse), via intranasal (i.n.) route, or FI-RSV at 1.875 μg/mouse via intramuscular (i.m.) route at day 0. Serum antibody titers against RSV were examined by ELISA (**A**), and the levels of induced serum neutralizing antibodies were assessed by immunoplaque assay (**B**) at day 21 post-immunization. The results for the neutralizing antibodies were expressed as the reciprocal of the highest serum dilution fold providing 50% inhibition of plaque formation. The induced isotype of RSV-specific IgG levels in BALB/c mice after immunization with rRSVs were assessed by ELISA (**C**), and ratios of IgG2a and IgG1 were calculated (**D**). nc: negative control group. Data are shown as mean ± SD. ns: no significance, *P < 0.05, **P < 0.01, ***P < 0.001.

To study the immunogenicity of the rRSVs, mice were immunized intranasally with 1 × 10^6^ pfu of rRSVs or *wt*RSV. Mice inoculated with PBS were used as negative control. Serum samples were collected 21 days after immunization, and the levels of RSV-specific serum IgG were detected by ELISA (Fig. 5A). The mice in both immunized groups produced significant RSV-specific IgG responses compared to the negative control (*P* < 0.001), but there was no difference between the IgG levels induced by the three rRSVs (*P* > 0.05). Compared with *wt*RSV, similar immunogenicity was observed for rRSV-Long/A2*cp* and rRSV-Long/A2*cptsΔSH* (*P* > 0.05). However, rRSV-Long/A2*cpts* was characterized by a slightly lower level of immunogenicity than that of *wt*RSV (*P* < 0.05).

The cross-protection against subgroup A *wt*RSV Long and subgroup B (WV VR1400) infection was also detected and the neutralizing antibody level was measured (Fig. 5B). Similar to the IgG responses, no significant difference in the levels of neutralizing antibodies was induced by immunization with the three rRSVs (*P* > 0.05). Analogically, when compared with *wt*RSV, similar neutralizing antibody responses and cross-protective effects were induced by rRSV-Long/A2*cp* and rRSV-Long/A2*cptsΔSH* (*P* > 0.05). However, rRSV-Long/A2*cpts* was characterized by a slightly lower response level (*P* < 0.05).

To determine the type of immune response induced in mice by rRSV immunization, the titers of IgG2a and IgG1, RSV-specific IgG subtypes, were examined, and the IgG2a/IgG1 ratio was calculated. The results showed that both rRSV and *wt*RSV immunization elicited a Th1-biased immune response, different from the Th2-biased immune response induced by intramuscular immunization with FI-RSV (Fig. 5C, 5D). In addition, the FI-RSV immunized mice also showed a significantly reduced neutralizing antibody response compared with mice in the *wt*RSV group (*P* < 0.01). However, the IgG response was similar between both FI-RSV and *wt*RSV immunization groups.

### The Efficacy of Intranasal Vaccination with rRSVs

**Fig. 6:**
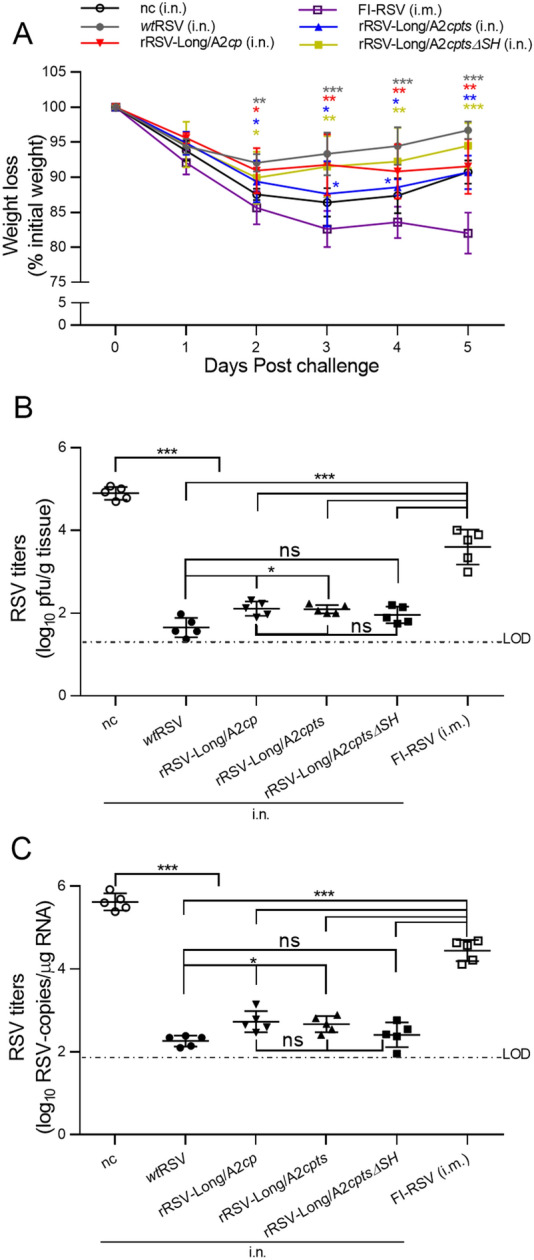
Induction of protective immunity against human respiratory syncytial virus (RSV) infection in the vaccinated BALB/c mice. After the immunized BALB/c mice were challenged with wild-type RSV (*wt*RSV, 1 × 10^6^ pfu/mouse) at day 28 post-immunization, the body weight changes were monitored daily from day 1 to day 5 following viral challenge (**A**). Lung homogenates were collected on day 5 post-challenge, and lung viral loads were determined by immunoplaque assay (**B**) and RT-qPCR (**C**). nc: negative control group. Data are shown as mean ± SD. ns: no significance, *P < 0.05, **P < 0.01, ***P < 0.001.

To evaluate the efficacy of RSV vaccine candidates, changes in body weight and lung viral titers were examined in vaccinated mice after RSV challenge. The mice were challenged with *wt*RSV 28 days after rRSV immunization, and their body weight was monitored for 5 consecutive days (Fig. 6A). Weight loss in challenged mice immunized by *wt*RSV and the three rRSVs was significantly reduced from day 2 to day 5 compared to that of FI-RSV-immunized mice (*P* < 0.05 or *P* < 0.001). On the other hand, mice immunized by either rRSV-Long/A2*cp* or rRSV-Long/A2*cptsΔSH* did not display significant differences in weight loss compared with that of *wt*RSV (*P* > 0.05), but a significant difference was observed between mice immunized with rRSV-Long/A2*cpts* and *wt*RSV on day 3 and day 4 after the challenge (*P* < 0.05). Moreover, the immunization with rRSV-Long/A2*cpts* resulted in weight changes different from those induced by rRSV-Long/A2*cp* or rRSV-Long/A2*cptsΔSH* at day 3 after challenge (*P* < 0.05, data not shown). The slightly decreased neutralizing antibody response observed in mice after immunization with rRSV-Long/A2*cpts* when compared to other rRSVs, mentioned above, may be an explanation for this phenomenon. Altogether, rRSV-Long/A2*cp* and rRSV-Long/A2*cptsΔSH* exhibited the best efficacy among the three rRSVs, which was reflected by weight changes after *wt*RSV challenge.

On day 5 after the challenge, the lung tissues were collected from the examined mice. Lung viral titers were analyzed by immunoplaque assay and RT-qPCR (Fig. 6B, C). Immunization with *wt*RSV and each rRSV resulted in decreased lung viral titers when compared to those observed in mice immunized with FI-RSV and mice from the negative control group (*P* < 0.001). On the other hand, there were no significant differences in lung viral titers among mice immunized with the three rRSVs (*P* > 0.05). However, the lung viral loads were significantly higher in mice from the rRSV-Long/A2*cp* and rRSV-Long/A2*cpts* groups than in the *wt*RSV-immunized group (*P* < 0.05). Further, no observable differences existed between the lung viral titers of mice immunized with rRSV-Long/A2*cptsΔSH* and *wt*RSV after *wt*RSV challenge (*P* > 0.05). These results showed that all rRSV-immunized mice were protected against RSV infection; however, rRSV-Long/A2*cptsΔSH* provided the best protection.

### Absence of Pulmonary Pathology and Eosinophilia in rRSV-Immunized Mice Following RSV Infection

**Fig. 7:**
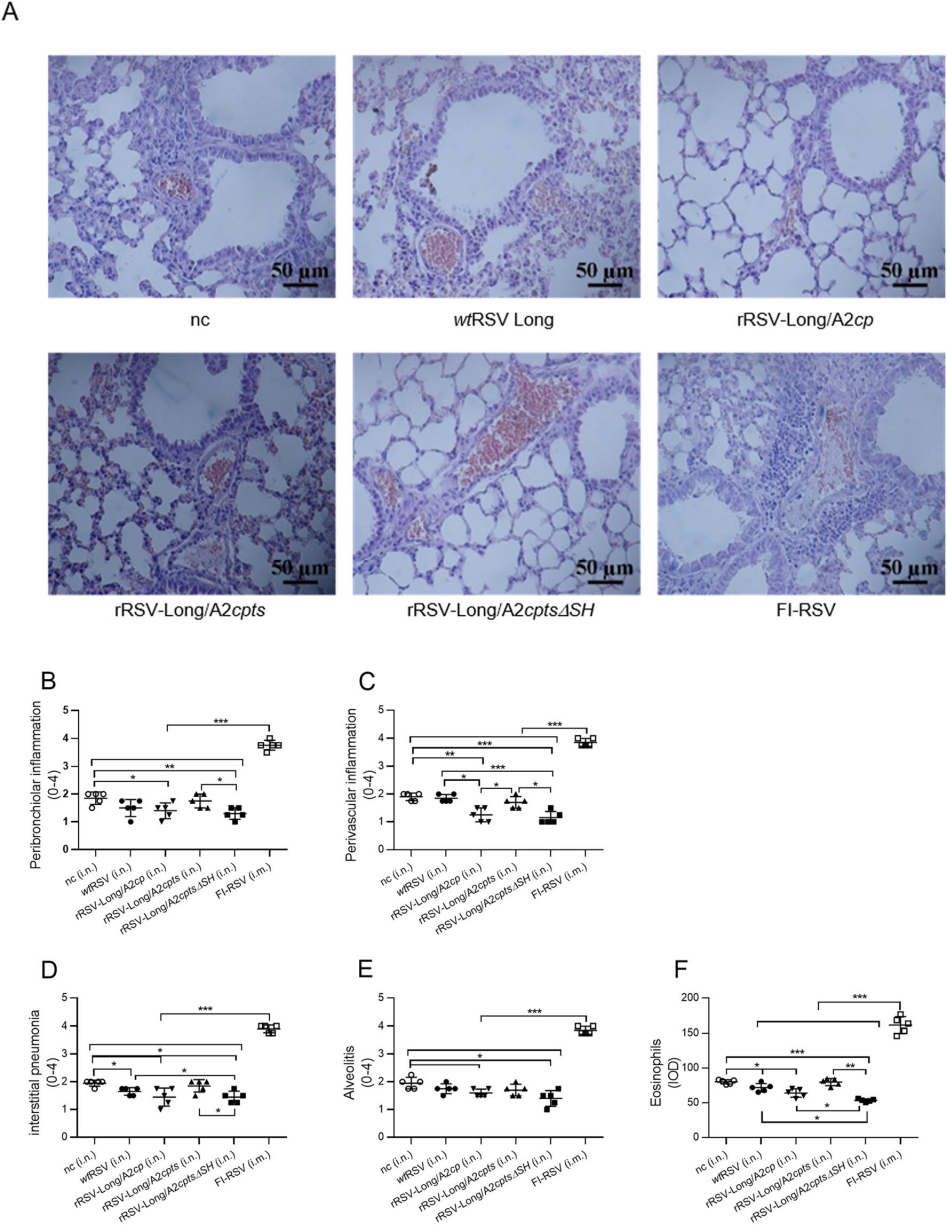
Absence of pulmonary histopathology and eosinophilia in mice vaccinated with recombinant human respiratory syncytial viruses (rRSVs) following RSV challenge. After the immunized BALB/c mice were challenged with wild-type RSV (*wt*RSV, 1 × 10^6^ pfu/mouse) at day 28 post-immunization, lung tissues were collected at day 5 post-challenge and pulmonary histopathology was analyzed by hematoxylin and eosin staining (H&E) (**A**). The H&E-stained lung sections from each mouse were scored for inflammation, including peribronchiolar inflammation (**B**), perivascular inflammation (**C**), interstitial pneumonia (**D**), and alveolitis (**E**). Distribution and intensity of eosinophils were measured and analyzed using specific stains and Image-Pro Plus (**F**). IOD: integrated optical density. nc: negative control group. Data are shown as mean ± SD. *P < 0.05, **P < 0.01, ***P < 0.001.

Since the FI-RSV vaccine causes ERD in immunized children and animals, the pathological examination of lungs in primed mice after RSV challenge is an important safety index for the RSV vaccine candidates. To this purpose, we collected the mouse lung tissues for histological sections 5 days after the challenge, performed H&E staining, and evaluated the pulmonary pathology under a microscope (Fig. 7A). The alveolar walls in mice immunized with FI-RSV were thickened, and alveolar cavities were compressed to form lung parenchyma. There was also a large number of inflammatory cells infiltrating around the blood vessels and bronchi. In negative control mice, the alveolar walls appeared thickened, the alveolar septa collapsed resulting in cavitation, and inflammatory cell infiltration was present around the blood vessels and bronchi. In the *wt*RSV- and rRSV-Long/A2*cpts*-immunized mice, thickening alveolar walls, cavitation, and increased levels of inflammatory cells were also observed in the lung tissues, albeit to a less extent. On the other hand, the immunization of mice with rRSV-Long/A2*cp* and rRSV-Long/A2*cptsΔSH* resulted in clear as well as less destroyed alveoli, and only a few inflammatory cells accumulated around the bronchi.

Lung sections from all mice were scored for inflammation around airways, blood vessels, and interstitial and alveolar spaces as described in the Materials and Methods (Fig. 7B–7E). A considerably severe (score around 4) lung histopathology was observed in mice vaccinated with FI-RSV while a less severe lung histopathology (score around 2) was characteristic for mice in the negative control group. The least severe lung histopathology (score range, 1 to 2) was observed in at least two mice in each rRSV-vaccinated group (rRSV-Long/A2*cp* and rRSV-Long/A2*cptsΔSH*) compared to the FI-RSV vaccination group (*P* < 0.001) and the negative control group (either *P* < 0.05, *P* < 0.01, or *P* < 0.001). These results showed that vaccination with two of the three rRSVs resulted in protective immunity without causing any obvious signs of ERD, while the documented FI-RSV immunization-triggered ERD after viral challenge was confirmed. Altogether, the observed pulmonary pathology exhibited similar trends to those of the relative body weight loss among the experimental groups.

In the lungs of mice immunized with FI-RSV we also observed the infiltration of eosinophils, unlike in the lungs of mice from the rRSVs and *wt*RSV vaccination groups (*P* < 0.001) (Fig. 7F). In particular, the lungs of mice immunized with rRSV-Long/A2*cptsΔSH* displayed the least number of eosinophils in comparison with the *wt*RSV vaccination group and the negative control group (*P* < 0.05; *P* < 0.01). This result is consistent with the histopathological observations, and indicates decreased inflammation owing to a better protection against RSV infection through immunization with rRSV-Long/A2*cptsΔSH*.

## Discussion

To our knowledge, this is the first report on the successful construction and rescue of rRSVs obtained from a parental strain other than A2 (parental strain Long) and bearing *cp*, *cpts,* or *cptsΔSH* mutations. Most of the cDNA-derived rRSVs are based on the *cp*RSV and *cpts*RSV parents, which are both biologically derived live attenuated viruses from the RSV A2 strain. Although differences exist in the genomic makeup as well as the encoded proteins (such as G protein) of *wt*RSV Long and RSV A2, both strains are classified as group A and genotype GA1 RSVs (Peret *et al*. 1998). Therefore, we aimed to examine the characteristics of RSV LAV candidates obtained from cDNA of the *wt*RSV Long parent rather than the biologically derived one, and used the rRSV-Long/A2*cp* parent, a counterpart of the biologically derived *cp*RSV.

The *att* phenotype and genetic stability are the critical features of live attenuated RSV vaccine candidates. We found that rRSV-Long/A2*cp* is non-*ts*, similar to the biologically derived *cp*RSV and rRSVA2*cp* (Crowe *et al*. 1994a, b, 1995; Whitehead *et al*. 1998b). Moreover, rRSV-Long/A2*cpts* and rRSV-Long/A2*cptsΔSH* share the same *ts* phenotype and shut-off temperature of around 37 °C, similar to their *cp*RSV derivative counterparts, *cpts*-*248/404* and rA2*cp248/404ΔSH* (Crowe *et al*. 1994a, 1995; Firestone *et al*. 1996; Karron *et al*. 2005; Lin *et al*. 2006; Schickli *et al*. 2012). We have monitored all the *att* mutation sites including the set of five *cp* mutations (Whitehead *et al*. 1998b) and *ts* mutations at the 248 and 404 *ts* markers in the serially passaged rRSVs from P1 to P9, and no reversion was observed at these sites.

However, during the temperature stress test of rRSV-Long/A2*cpts*, all the randomly selected *tsi* viruses had nt changes causing reversion to the wild-type amino acid or nt at the 248 or 404 *ts* markers. According to a previous *in vitro* analysis of MEDI-559 from MedImmune, an updated version of rA2*cp248/404/1030ΔSH* and containing 39 silent nt substitutions (including TTA to CTG substitution at the 248 *ts* marker), MEDI-599 displayed a similar nt change at the 248 site as the resultant *tsi* viruses (Schickli *et al*. 2012). In contrast to our observation, no changes were observed at the 404 *ts* marker located in MEDI-559 and no MEDI-559-infected plates showed positive RSV immunostaining at 38 °C. This phenomenon may be attributed to both the lower shut-off temperature of MEDI-559 (35 °C) and the lower passage temperature (37 °C) used in the cited study than the 39 °C passage temperature applied here for rRSV-Long/A2*cpts*. Although no changes were reported at the MEDI-559 404 *ts*, reversion at the 404 locus is possible and did occur during the earlier clinical trials with *cpts248/404* in young infants (Whitehead *et al*. 1998a; Wright *et al.* 2000). In our analysis of nt changes at the *ts* markers, cDNA from only five randomly chosen positive wells was sequenced and analyzed for the presence of gene fragments containing *ts* mutations. Despite its convenience, our method is not as comprehensive and accurate as high-throughput deep sequencing of the whole genomes. Therefore, further analysis might be needed to uncover some of the mutations.

The characteristics of the *in vivo* *att* phenotypes of rRSVs were uncovered by infecting mice intranasally. Compared with *wt*RSV, all the three rRSVs had lower replication activity *in vitro* (Fig. 2 and Fig. 3), indicating that the *cp* mutation endowed all the three rRSVs with *att* phenotype from ‘host-range’ restrictions functioning only *in vivo* but not *in vitro*, similar to *cp*RSV in chimpanzees and humans (Whitehead *et al*. 1998a, b). Furthermore, the titers of the two rRSVs with *ts* mutations were reduced in the lower respiratory tract compared with those in the upper respiratory tract (Table 1), in sharp contrast with the titers of rRSV-Long/A2*cp.* These results demonstrate that restricted replication at physiological temperatures is attributed to the *ts* phenotypes of rRSV-Long/A2*cpts* and rRSV-Long/A2*cptsΔSH*. Moreover, similar levels of serum IgG and neutralizing antibodies were found among mice vaccinated with rRSV-Long/A2*cp*, rRSV-Long/A2*cptsΔSH*, and *wt*RSV (*P* > 0.05). However, despite the shared *ts* phenotype, rRSV-Long/A2*cpts* immunization, unlike rRSV-Long/A2*cptsΔSH* immunization, resulted in a slightly decreased antibody response compared to *wt*RSV immunization (*P* < 0.05). This result slightly differs from that of a previous report (Whitehead *et al*. 1999), possibly owing to an increased transcription of the downstream neighboring genes *G* and *F* in *ΔSH* rRSV (Bukreyev *et al*. 1997). Notably, despite the inconsistency in comparison with *wt*RSV, there were no significant differences in IgG and neutralizing antibody responses among the three rRSVs, similar to the previous observations in chimpanzees (Whitehead *et al*. 1999).

The most dramatic reduction of lung viral loads occurred in mice vaccinated with rRSV-Long/A2*cptsΔSH* among the three rRSVs (*P* < 0.05). Similar results were observed for *wt*RSV-immunized mice. Therefore, compared with the other two rRSVs, rRSV-Long/A2*cptsΔSH* exhibited the best protective properties against RSV infection. Finally, analyses of lung pathology and eosinophilia in mice after viral challenge showed the absence of ERD following immunization by either of the rRSVs, especially by rRSV-Long/A2*cptsΔSH*. These results are likely attributed to the potent efficacy of rRSV-Long/A2*cptsΔSH* immunization. FI-RSV immunization is known to be followed by ERD; however, there are still uncertainties regarding the underlying mechanism of this phenomenon. Generally, the key factors are thought to be the poor functional activity of the induced RSV-specific antibodies and a Th2-biased CD4 T-cell response characterized by increasing cytokine levels. Previous studies have already confirmed that ERD does not occur after a natural RSV infection or inoculation with RSV LAV candidates (Wright *et al*. 2007), consistent with our results.

Altogether, rRSVs bearing either *cp*, *cpts,* or *cptsΔSH* mutations have been successfully constructed and rescued from a parent strain other than A2 (*wt*RSV Long). These data indicate that the proposed strategy allows to develop promising RSV LAV candidates, with *att* phenotype, immunogenicity, genetic stability, and safety similar to those characteristic for strains derived from the traditional RSV A2 parent. Although it is necessary to confirm these data in animal models more susceptible to human strains of RSV, such as cotton rats, which are 100-fold more permissive than BALB/c mice (Prince *et al*. 1978, 1999; Byrd and Prince 1997; Taylor 2017), our work has paved the way to improve the safety, immunogenicity, and efficacy of live attenuated RSV vaccine candidates derived from the *wt*RSV Long parent.

In summary, we have successfully constructed and rescued three rRSVs bearing *cp*, *cpts,* or *cptsΔSH* mutations based on a reverse genetics technology. We used the *wt*RSV Long parental strain, which differs from the standard RSV A2 strain. However, the three cDNA-derived rRSVs exhibited biological and immunological similarities with their RSV A2 counterparts. Moreover, the results of *in vitro* and *in vivo* experiments showed that the rRSVs with the introduced mutations (*cp, 248ts,* and *404ts*) from the RSV A2 strain exhibited optimal levels of attenuation, efficacy, and genetic stability, which could be further improved when combined with the *SH* gene deletion. Therefore, we can preliminarily conclude that the development of live attenuated RSV vaccines with RSV Long as parent strain is a promising strategy which needs to be further explored in the future.

## Supplementary Information

Supplementary file1 (PDF 169 kb)
