# Supplementary material for: Evaluation of the Safety and Immune Efficacy of Recombinant Human Respiratory Syncytial Virus Strain Long Live Attenuated Vaccine Candidates
Source: Virol Sin. 2021 Feb 9;36(4):706–20. doi: 10.1007/s12250-021-00345-3 (PMC8379332; doi:10.1007/s12250-021-00345-3)
Supplement: Supplementary file 1 — Supplementary file1 (PDF 169 kb) [file 12250_2021_345_MOESM1_ESM.pdf]

VIROLOGICA SINICA

Electronic Supplementary Material

Evaluation of the Safety and Immune Efficacy of Recombinant Human Respiratory Syncytial Virus Strain Long Live Attenuated Vaccine Candidates

Li-Nan Wang[1] • Xiang-Lei Peng[1] • Min Xu[1] • Yuan-Bo Zheng[1] • Yue-Ying Jiao[1] • Jie-Mei Yu[1] • Yuan-Hui Fu[1] • Yan-Peng Zheng[1] • Wu-Yang Zhu[2] • Jin-Sheng He[1]✉

1. College of Life Sciences and Bioengineering, School of Science, Beijing Jiaotong University, Beijing 100044, China
2. National Institute for Viral Disease Control and Prevention, Chinese Center for Disease Control and Prevention, Beijing 102206, China

Supporting information to DOI: 10.1007/s12250-021-00345-3

Table S1 The primers used for amplifying DNA copies encompassing the encoded attenuation (*att*) mutations from recombinant RSVs (rRSVs)

Gene fragment bearing the indicated attenuated mutation	Primer sequence
N 799 G>A	F: 5′-GATCTGGTCTTACAGCCGTGA-3′
	R: 5′-TGAGTTGTTCAGCATATGCC-3′
F 653, 654 AA>CG	F: 5′-GCACAACAGCAGCAAACAATCGA-3′
	R: 5′-TCACAGTACCATCCTCTGTCAG-3′
F 1568, 1569 CA>TT	F: 5′-GTGGACACTGTGTCTGTAGGTA-3′
	R: 5′-TTGCAAGGATTCCTTCGTGACA-3′
L 956, 957 GT>AC	F: 5′-TGCAGCATCCTCCATCATGGT-3′
	R: 5′-GCATCTGTGATGTTGTTGAGCA-3′
L 5068, 5070 CT>TC	F: 5′-AGCCAAGATGCAAGTTTACA-3′
	R: 5′-TGGCTGTATTACCTGAATGATC-3′
M2 9 T>C	F: 5′- AGCACACCAGTCACACTAAGCA-3′
	R: 5′- TTGCTCATGGCAACACATGCTG-3′
L 2491, 2492 CA>TT	F: 5′- GTAGTGATGTGCTGGATGAACT-3′
	R: 5′- AACAAGTTGGGATCACCACCAC-3′

Table S2. The genetic stability of rRSVs evaluated by the sequenced DNA copies surrounding the attenuation mutations.

rRSVs	The nucleic acids for the attenuation mutations						
	Val-267-Ile/N ATT	Glu-218-Ala/F GCG	Thr-523-Ile/F ATT	Cys-319-Tyr/L TAC	His-1690-Tyr/L TAC	Gln-831-Leu/L TTA	T-7605-C/M2 AAC
rRSV-Long/A2*cp*	ATT	GCG	ATT	TAC	TAC	CAA	AAT
rRSV-Long/A2*cpts*	ATT	GCG	ATT	TAC	TAC	TTA	AAC
rRSV-Long/A2*cptsΔSH*	ATT	GCG	ATT	TAC	TAC	TTA	AAC

The nucleic acids for the attenuation mutations in recombinant viruses of rRSV-Long/A2cp, rRSV-Long/A2cpts, and rRSV-Long/A2cptsΔSH sequenced every other generation until P9.

Table S3. Changes and their frequencies in the L and M2 genes of biologically derived RSV *tsi* viruses.

Virus	248 *ts* marker aa 831 in L	404 *ts* marker nt 9 in M2 gene-start	Frequency
*wt*RSV	Gln, CAA or CAG	T	N/A
rRSV-Long/A2*cpts*	Leu, TTA	C	N/A
tsi revertants of rRSV-Long/A2*cpts*	Leu, TTA	T	1/5
	Gln, CAA	C	4/5

N/A: not applicable; *tsi*: temperature sensitive intermediate; *ts*: temperature sensitive; aa: amino acid; nt: nucleotide.
